# Bodily Expression Support for Creative Dance Education by Grasping-Type Musical Interface with Embedded Motion and Grasp Sensors †

**DOI:** 10.3390/s17051171

**Published:** 2017-05-20

**Authors:** Tomoyuki Yamaguchi, Hideki Kadone

**Affiliations:** 1 Department of Intelligent Interaction Technologies, University of Tsukuba, Tsukuba 305-8573, Japan; 2 Center for Innovative Medicine and Engineering, University of Tsukuba, Tsukuba 305-8577, Japan; kadone@md.tsukuba.ac.jp

**Keywords:** creative dance, supporting dance education, grasping-type interface, embedded sensing device, motion analysis

## Abstract

Dance has been made mandatory as one of the physical education courses in Japan because it can cultivate capacities for expression and communication. Among several types of dance education, creative dance especially contributes to the cultivation of these capacities. However, creative dance requires some level of particular skills, as well as creativity, and it is difficult to presuppose these pre-requisites in beginner-level dancers without experience. We propose a novel supporting device for dance beginners to encourage creative dance performance by continuously generating musical sounds in real-time in accordance with their bodily movements. It has embedded sensors developed for this purpose. Experiments to evaluate the effectiveness of the device were conducted with ten beginner-level dancers. Using the proposed device, the subjects demonstrated enhanced creative dance movements with greater variety, evaluated in terms of Laban dance movement description. Also, using the device, they performed with better accuracy and repeatability in a task where they produced an imagined circular trajectory by hand. The proposed interface is effective in terms of creative dance activity and accuracy of motion generation for beginner-level dancers.

## 1. Introduction

Dance is a bodily action intended to convey to others the imagination conceived by the dancer. In Japan, dance has been a compulsory subject for elementary school since 2011, for junior high school since 2012, and for high school since 2013, following revisions to the curriculum guidelines in 2008 by the Ministry of Education, Culture, Sports, Science and Technology (MEXT). The dance in compulsory education is composed of Creative Dance, Folk Dance, and Hip Hop as a modern dance [1]. In particular, the improvisational aspects of creative dance are expected to contribute to the cultivation of expressive ability, creativity, and imagination. Communication skills are also expected to be cultivated by sharing imagination with others through bodily expressions and dance improvisation actions. Dance education is useful not only for students under compulsory education but also for college students and adults with no experience of the compulsory education of dance. For the latter, it can provide an opportunity for them to acquire basic social capabilities such as creativity, faculty, and the ability to grasp a situation by performing creative dance [2].

However, the improvisational skills required in creative dance are difficult to be presupposed in beginner-level dancers, because the skill required to convert their imagination to bodily expression necessitates a certain level of experience. Improvisatory dance allows a tremendous variety of motion, which makes it different from other types of dance, such as rhythm or folk dancing, where motion is chosen from a set of predefined motion repertoires based on a melodic and rhythmic stream of music.

Concerning this difficulty for beginners, a report [3] by National Institute for Educational Policy Research in Japan proposed four kinds of criteria to guide the development of creative dance by beginner-level dancers: Involvement (liveliness of motion with rhythm and tempo without hesitation or timidity), Creativity (variety of created motions that follow a given theme), Motor Skill (expressiveness of entire body motion including hands and fingers), and Knowledge (knowledge and understanding of theme, dance performance, and exercise). In this context, Terayama and Hosokawa [4] investigated conventional teaching methods for improvisation dance and identified the usefulness of “prompts”. They reported that educators in the field use common prompts to inspire the improvised expression of learners immediately prior to dancing. The educators indicate imagination by displaying playing cards or providing motor tasks to ease the conversion of imagination into bodily expressions. From this perspective, we can consider the necessity and importance of a kind of support that can continuously prompt to derive and encourage the imagination of bodily expression and the ability to create expressive motions.

Because these educational indications were given in conceptual terms, they tended to be susceptible to the subjective impression of the teachers [1]. Conversely, Miyamoto [5] focused more on describing the detail of bodily motion, such as amplitude, smoothness, continuity, and variety in velocity and strength of motion of each body part for the evaluation criteria in education of creative dance. Translating the educational points of the previous section into motion description, the problem in bodily motion for beginner-level dancers is primarily that the motion tends to be small and less variate and accompanied with hesitated and bewildered initiation.

Grasping is another important aspect of bodily motion in creative dance. It represents subtle finger motion that must be developed in beginner-level dancers according to the above educational guideline. Further, even when the fingers seem to be stationary, the force given by the fingers represents internal emotion and therefore devotion to a theme [6,7], which is unobservable from outside.

Recent literature in neuroscience reports on modulation of sensory integration in the brain induced by real-time auditory feedback of motion information. Research on the effect of a sensor glove [8], which presents the tactile information of hand by sound, investigates the brain areas that are responsible for the modulated sensory integration. Behavioral investigation on the effect of auditory feedback of footstep during gait [9] reported significantly larger steps and forward drift of the body on a treadmill induced by the feedback, and discussed the changes in the mechanism of self-motion perception and sensory-motor integration. These neurological and behavioral evidences suggest that sound feedback of self-motion can be a useful tool for enhancing dance movement.

Based on these perspectives, to support bodily expression of creative dance in beginner-level dancers, in this paper we propose a device that provides prompts that reflect the relevant motion and grasp parameters to induce and reinforce creative dance motions. This paper presents (1) a supporting interface that triggers and senses the dancer’s motion; (2) a sound feedback mechanism used to enhance the expression during dancing; and (3) quantitative evaluation of the dance expression including grasping that indicates the effectiveness of the proposed method.

**Figure 1:**
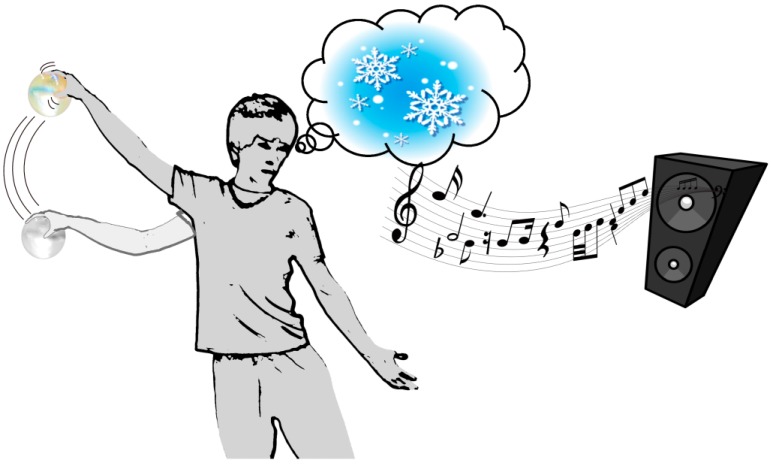
Conceptual diagram for the creative dance support of the proposed grasping-type interface. Motion and grasping force sensed by the interface are presented back to the user in real-time as a variation in musical sound. The sound encourages the user to generate motion continuously and accurately following the imagined trajectory. It also helps the user to conceive imagination for the next motions.

In the proposed method, we develop a supporting interface for beginners that generates prompts for bodily motion during creative dance performance as displayed in Figure 1. This interface has three features: it is ball-shaped, wireless, and hand-held. Because of these features, the interface can be held in the user’s hand during a dance performance, allowing them free motion and detecting the grasping motion. The interface controls sound parameters such as the note, volume, and tempo according to variations in the grasping force and bodily motion. The sound is then presented back to the user in real-time. Because of this feedback, users can understand the association between sound and their bodily and grasping motions. Utilizing the imagination to generate sound sequences, users can be prompted for successive dance motions. In this manner, the sounds can continuously support the user’s successive creative performance. In the experiments to verify the effectiveness of the interface, we focus on the performance of beginner-level dancers during creative dance with the interface. Their creative dance performance is evaluated based on Laban Movement Analysis (LMA) [10,11] and the amount of variation in the grasping force. The accuracy of the realization of imagined motion is evaluated in additional experiments.

In our pilot study of this experiment with fewer subjects, a positive result was obtained [12]. In [12], order effect was not considered and the evaluation depended simply on the average and standard deviation of accelerometer data. In contrast, in this paper, the results are evaluated according to Laban motion standard based on the abovementioned standpoints, and order effect is dealt with by introducing reversed order of the conditions with more number of subjects. Moreover, the second experiment, that investigates motion accuracy to evaluate the accuracy of physical representation of motion image under the support of the device, is newly presented here. Both the real-time creation of motion image and the physical representation of the created image are the essential and inevitable targets of support in supporting creative dance. These two experiments combined can guarantee the effectiveness of the interface. Also, for both of the experiments, statistical validation of the results is added. These results prove the effectiveness of the proposed interface, at the level of its realistic and broad application to beginner level dancers.

The remainder of this paper is organized as follows: Section 2 describes related research. Section 3 presents the proposed bodily expression support interface that includes the hardware design, control methods, and performance styles. Section 4 provides the experiment settings. Section 5 presents the experiment results and discussion that confirms the usability of the proposed interface and explores the effect of the proposed grasping interface on the creation of a musical sound-space. Section 6 presents the conclusions.

## 2. Related Works

Since dance courses have become compulsory, supporting systems for dance education have gained importance. Among the studies to support dance improvement, Sato et al. [13,14] focus on arm movements in street dance to analyze the movements of novice and expert dancers. These studies evaluate from the perspective of motion analysis, whereas conventional approaches evaluate movement through sensibilities such as aesthetic and artistic points in dance performance. These studies focus on observing the difference between the movements of novices and experts and evaluate the characteristics of expert dancers; however, this research has not been considered to improve the dance performance for initial beginner-level dancers.

There have been other studies to evaluate motion using motion capture systems with virtual reality technologies [15,16,17,18]. These studies are useful in the training of motions with trajectories already determined such as ballet. It is possible to use these to improve the accuracy of creative dance; however, these systems do not encourage creative performance for beginners. Tadenuma et al. [19] also employed virtual reality technology to analyze dancer movements using Kansei information processing technology. They developed a system for transmitting to others, through visual images, the intent of a dancer estimated by the physical features in a video. However, because the line of sight faces the display system, free dance expression is restricted. Moreover, the dance space is limited because of the system required sensing areas for the motion capture system.

There is also research that proposes a new dance technique through lighting representation (LED Lighting System) [20]. This study evaluates the effects of lighting representation on physical expression; however, this is also for experts, rather than novices. Unlike display systems, this approach employs wearable LEDs. Because there are many parameters to determine the light pattern, it is necessary to choreograph the light according to the motions in advance; this does not correspond to a real-time dancing operation.

There have been proposals that use portable devices such as haptic interfaces and mobile phones. This line of research is particularly active in musical application [21,22,23]. These studies introduce various types of sensing technique to detect human motion and the measured body movements are mapped to music or sound. These performance systems liberate a performer from the usual physical limitations and provide different capabilities for music creation. Unlike the methods of confirming a dance by visualization, this is called sonification technology and has been used by professional dancers and artists [24,25]. There are many wearable devices; however, these are not suitable for usage to support dance beginners in an environment with multiple people because the installation tends to be complicated. Therefore, an interface that is portable and does not include dance field restrictions is necessary. Mobile phones, including iPhones, have become useful devices because of their multiple sensors that can be used to detect human actions and their desirable sizes. Many software applications have been developed to perform music production and editing [26,27]. However, such applications focus on the development of a musical interface to create novel music. These instruments require both hands, and are therefore not suitable for facilitating free-dance performance.

Thus, for beginners’ dance support, it is important that the system can address multiple people via a non-wearing approach, include the possibility to measure the movement of the dancer without limiting the dance space, and function in real-time. In this research, we propose a method to perform musical feedback using a grasping-type portable interface.

## 3. Proposed System

### 3.1. Design

**Figure 2:**
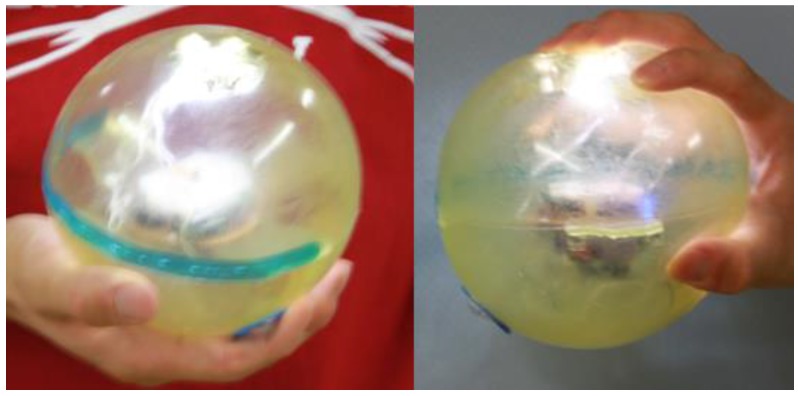
Grasping-type musical interface, TwinkleBall.

**Figure 3:**
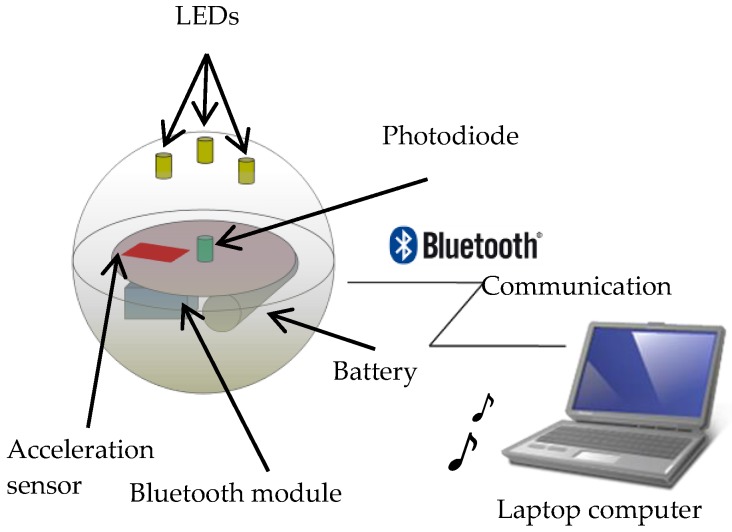
Overview of the proposed system. The proposed system contains acceleration, Bluetooth module, Photodiode, LEDs, and Laptop computer.

The design of our musical interface, TwinkleBall [28,29], is presented in Figure 2. The main body of the proposed interface consists of a rubber ball, Bluetooth wireless communication module, photodiode, three-axis accelerometer, LEDs, peripheral interface controller (PIC), and battery (9.0 V). All the electronic devices are enclosed in the rubber ball, which is translucent and hollow. The measurement range of the acceleration sensor is ±3.6 g. The peak wavelength of the photosensor is 560 nm. The Bluetooth module, photosensor, three-axis sensor, PIC, and battery are placed on an electronic circuit board inside the core, which is affixed to the rubber ball using rubber sheets. To convert the analogue signal of the sensors into a digital signal, 10-bit A/D conversion is used. The sampling rate of the A/D converter is 550 Hz. The energy autonomy of the device is 1 h. The LEDs are placed on the interior surface of the rubber ball. The specifications of the rubber ball are as follows: diameter, 152 mm; mass, 260 g; material, polyvinyl chloride (PVC). Figure 2 indicates that performers and audiences can easily see TwinkleBall, which shines by virtue of its LEDs and translucent material, even if the performance is staged under low-light conditions. As indicated in Figure 3, the signal output from the photosensor and accelerometer are digitized and sent to an external computer via the Bluetooth wireless communication module. The interval of the communication is set at 35 ms.

### 3.2. Sound Generation Mechanism

**Figure 4:**
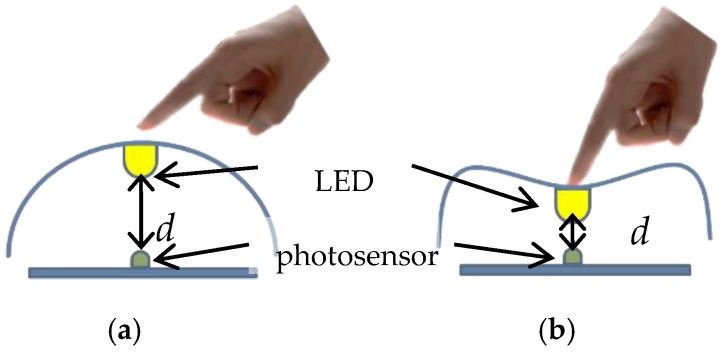
Difference between cases with and without force: (**a**) Case without force; (**b**) Case with force.

To apply the proposed interface to a dance performance, it is important for the generated sounds to represent bodily motions. In this study, we design the sound application such that the grasping motion controls the note and the moving motion with the interface controls the volume and tempo. We use MIDI sounds as output. In particular, when the shape of the rubber ball changes because of the users’ grasping force, the distance *d* between the internal photosensor and LEDs varies as illustrated in Figure 4. Because the illumination intensity is inversely proportional to distance *d*, changes in the grasping force produce different output signals from the photosensor. This output signal is sent to the computer via the Bluetooth module and the note is then tuned based on its value. Because we use MIDI sounds, the range of the note is seven bits. The 10-bit digital signal from the photosensor is normalized into 7-bit before the MIDI output is calculated. Let *P_min_* and *P_max_* be the illumination intensity at the maximum distance of *d* and the minimum distance of *d*, respectively, and *p* be the input of the illumination intensity. Then the note is calculated as follows:

(1) $$note=\;n_{a}+n_{range}\cdot \left(\frac{p-P_{min}}{P_{max}-P_{min}}\right)$$

where *n_a_* is the reference value of note. As the range of MIDI note is 0 to 127, *n_a_* is set as 60 which is C4 (Middle C) in scientific pitch notation with a frequency of 261.6 Hz. *n_range_* is set as 24. We can control 2 octaves from 60 to 84.

**Figure 5:**
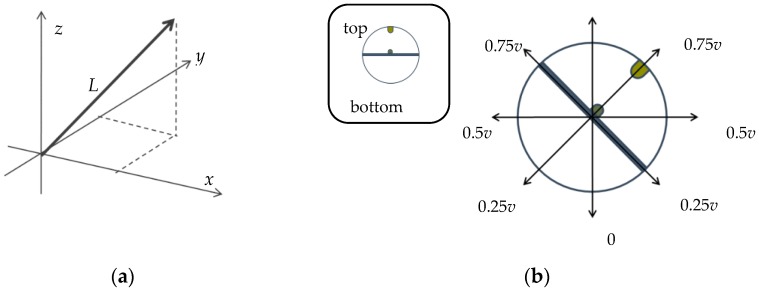
Volume control: (**a**) Vector length; (**b**) Static case.

The proposed interface can change sound volume. The measurement value from the acceleration sensor changes when the dancers move the grasping interface through their motions. The accelerometer measures acceleration with respect to the *x*, *y*, and *z*-axes. For noise reduction of accelerometer, we applied a smoothing filter which is based on moving window average of 10 samples. The acceleration values are sent to the computer via the Bluetooth module. The computer calculates three-dimensional (3D) acceleration vector length *L* using these values and the volume is determined by this length as indicated in Figure 5a. The sound volume range is also seven bits, which corresponds to the resolution of the MIDI velocity. Therefore, the calculated vector is normalized to correspond to this range. The volume does not depend on the direction of movement because we simply use vector length *L*. Volume control is divided into two cases, namely, static and dynamic. In the dynamic case, where dancers move the interface through their motion, the volume is calculated linearly. In the static case, where dancers do not move, yet grasp the interface, the volume depends on the gradient angle of the interface. Although the interface shape is a sphere, it is divided into top and bottom hemispheres. The volume is determined as indicated in Figure 5b. In this paper, *v* is set to 60, which is almost half the range of the MIDI velocity determined by exploration in the preliminary experiments. Figure 5 illustrates the case of a 45-degree angle; this corresponds to a volume of 0.75*v*. Then, *L* is calculated as follows:

(2) $$L=\;\sqrt{{\left(x-x_{i}\right)}^{2}+{\left(y-y_{i}\right)}^{2}+{\left(z-\frac{x_{i}+y_{i}}{2}\right)}^{2}}$$

(3) $$L_{z}=\;\sqrt{{\left(\frac{z-z_{i}}{2}\right)}^{2}}$$

where, *x*, *y*, and *z* are the sensed acceleration values. *x_i_*, *y_i_*, and *z_i_* are initial offsets of the sensor values which are measured before a user conducts dance, with the ball interface in a stationary state with the *z*-axis aligned with the vertical. *L_z_* is used for the static case. Finally, we calculate the volume as:

(4) $$volume=\;\{\begin{array}{cc} L_{z}\cdot v & if\;g-C\leq L\leq g+C\;\left(static\;case\right)\;\\ L\cdot v & otherwise\;\left(dynamic\;case\right) \end{array}$$

where, *C* is a threshold value to deal with sensing noises, and *g* represents gravity. By Equation (4), the range of volume is 0 to 60 in the static case and 60 to 127 in the dynamic case, as the range of MIDI velocity is 0 to 127.

The proposed interface can also control the tempo to realize numerous expressions. However, all the sensing data from the interface are used to control the note and volume. Therefore, we employ the time sequence data of vector length *L* to change the tempo. We calculate the average value of *L* as follows:

(5) $$k=\frac{\sum_{i}^{n}L_{i}}{n},$$

where *k* is the average value of *L* and *i* is the communication index. In this paper, *n* is set at eight because the interval of communication between the interface and the laptop is 35 ms. Then, the tempo is calculated by the following step function:

(6) $$tempo=\{\begin{array}{cc} \begin{array}{c} 1600,\\ 800,\\ \begin{array}{c} 400,\\ 200, \end{array} \end{array} & \begin{array}{c} if\;k\leq T\\ if\;T<k\leq 2T\\ \begin{array}{c} if\;2T<k\leq 3T\\ otherwise \end{array} \end{array} \end{array},$$

where *T* is a threshold value to create the step function. In this paper, *T* is set at 2*v*. The tempo is not changed in the static case; however, it is changed through human motion in the dynamic case.

**Figure 6:**
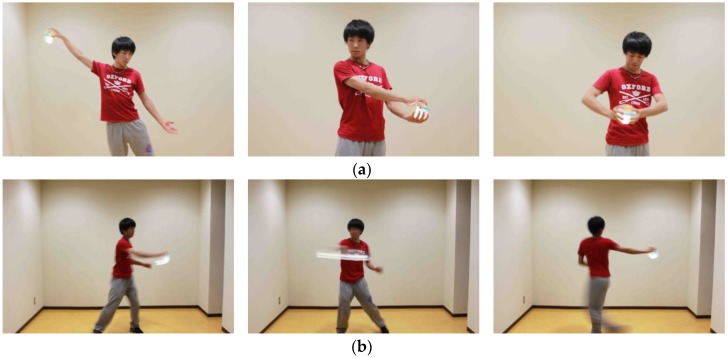
Scenes of musical performances using TwinkleBall: (**a**) Grasping motions; (**b**) Moving motions.

Figure 6 displays scenes of a dance performance where the proposed interface is used. Figure 6a depicts the dancer changing the note by varying the grasping force with a single hand or both hands. Figure 6b depicts the dancer varying the volume and tempo by moving in a large motion such as waving. The proposed system is not sensitive to intricate motions; however, the strength of the dance movement influences the volume and tempo control. Therefore, the system responds to different dance motions.

## 4. Experiments

In this section, we describe the experiments performed using TwinkleBall to confirm the validity of the proposed approach. We performed two experiments; the experiments focused on the improvement of creative activity and movement accuracy. Ten male and female subjects (university students and researchers) participated in the experiment. None of the subjects had previous dance education. All the subjects provided written informed consent to participate in these experiments.

### 4.1. Creative Activity Experiment

#### 4.1.1. Objective

We performed this experiment of dance improvisation using TwinkleBall with beginners as the subjects. For these subjects, this was the first time they performed an improvisation dance. The objective of this experiment was to confirm that TwinkleBall can support the expression of beginner-level dancers in creative dance. We evaluated the effectiveness of the sound generated by TwinkleBall by comparing the motion data between the conditions of “sound” and “mute.” In the “sound” condition, users held TwinkleBall in their hand while dancing and TwinkleBall generated sound that represented the motion. In the “mute” condition, TwinkleBall was muted and did not generate sound.

#### 4.1.2. Setting

The dancing space for the experiment was set at 2.5 m × 2.5 m. The procedure for the experiment was as follows:

Step 1: Explanation
  First, we explained the specifications of TwinkleBall. In the MIDI, there were 128 possible program sounds as the defining musical instrument sounds. Each subject selected a sound number for the output sound to be used with his/her motions.
Step 2: Creative dance theme
  Each subject considered a theme for the creative dance.
Step 3: Step Dance performance
  The subject danced twice: (1) the subject grasped TwinkleBall without sound (i.e., mute TwinkleBall), (2) the subject grasped TwinkleBall with sound. We observed one minute per dance. The theme (from Step 2) chosen by the subjects was the same in both dance experiments. To reduce order effects, we conducted experiments with two groups of five people. Half of the subjects received (1) first followed by condition (2); the other half received condition (2) first followed by condition (1).
Step 4: Step Completing questionnaires
  Finally, the subjects completed questionnaires orally for a qualitative evaluation. The questions were as follows:
  Q.1 Do you think you could dance along to the theme without sound?
  Q.2 Do you think you could dance along to the theme with sound?
  Q.3 Did your motion and sound correspond when there was sound?
  Q.4 Was the sound by TwinkleBall useful to determine successive motions while dancing?
  Q.5 Did you feel TwinkleBall restrained your dance?

The score was chosen from a range of five (positive) to one (negative) per question.

#### 4.1.3. Evaluation Methods

To investigate the experiment data, we calculated the values for both the “sound” and “mute” conditions. We employed Laban Movement Analysis (LMA) [10,11] for the evaluation of all the dance movements including the force of bodily motions, changes in bodily motions, and the calculation of the standard deviation of the grasping motion for evaluation of the hand action, which is a fine movement expression.

The LMA system was developed for describing, interpreting, and documenting a variety of human movement throughout the entire body. This is a useful method to evaluate the motion quantitatively [23,24,25,26,27,28,29,30]. In this paper, we used Weight Effort for strength of dance and Time Effort for briskness of dance.

Weight Effort denotes the strength of the bodily motion of creative dance. We calculate the force per unit time during the dancing experiment, *We*, as follows:

(7) $$We=\;\frac{\sum^{\text{​}}ma}{t},$$

where *m* is the mass, *a* is the same as *L* from Equation (2), and *t* is the time duration of the experiment. In this experiment, *m* is constant (i.e., *m* = 1) and *a* is collected from the acceleration sensor.

Time Effort denotes the briskness of the change in bodily motions. This is an index to evaluate the characteristics of the sudden movement of dance, which corresponds to jerk. We calculate the jerk per unit time, *Wt*, as follows:

(8) $$Wt=\;\frac{\sum \frac{da}{dt}}{t},$$

For the evaluation of the fine grasping motion, we measured the lighting intensity, *d_l_*, and calculated its standard deviation, *Wg*, as follows:

(9) $$Wg=\;\sqrt{\frac{\sum^{\text{​}}{\left(d_{l}-d_{ave}\right)}^{2}}{n}},$$

where *n* is the element count and *d_ave_* is the moving average value of *d_l_* per unit time.

Based on the above three evaluation methods, we evaluated the overall motion of the dance and fine grasping motion.

### 4.2. Movement Accuracy Experiment

#### 4.2.1. Objective

The objective of the second experiment was to confirm that the subjects were moving their body according to their imagined motion. In this experiment, the movement task was to perform unconstrained circular hand motion at a constant speed while maintaining the acceleration amplitude at one of three designed constant values (low, 10 m/s^2^; middle, 14 m/s^2^; high, 18 m/s^2^). The effectiveness of the sound generated by TwinkleBall was evaluated by comparing the motion data between the conditions of “sound” and “mute.”

#### 4.2.2. Setting

**Figure 7:**
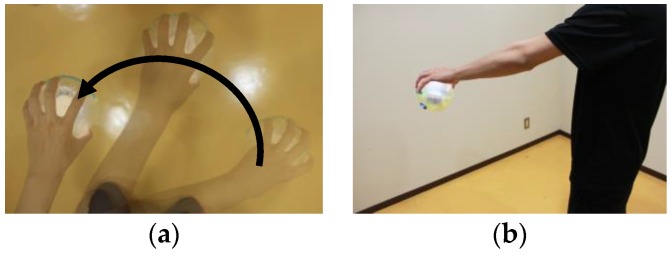
Experimental setup for circular hand motion: (**a**) Top view; (**b**) Side view.

The experimental setup is illustrated in Figure 7. The procedure for the experiment was as follows:

Step 1: Adjustment to target speed
  The subjects performed circular hand motions and adjusted until achieving the target acceleration (low, 10 m/s^2^; middle, 14 m/s^2^; high, 18 m/s^2^).
Step 2: Maintaining target speed
  After confirming that the target acceleration was reached, the experimenter requested the subjects to maintain the hand motion constant for five seconds. The subjects performed hand motion twice for each of the three accelerations: (1) subject grasped TwinkleBall without sound (i.e., mute TwinkleBall) and (2) subject grasped TwinkleBall with sound. To reduce order effects, we conducted the experiments with two groups of five people. Half of the subjects performed (1) first followed by condition (2); the other half performed condition (2) first followed by condition (1).

Steps 1 and 2 were repeated for each of the three accelerations.

#### 4.2.3. Evaluation Method

In this experiment, the movement task was to perform uniform circular hand motion. The centripetal acceleration measured by the acceleration sensor in the interface must be constant to achieve a uniform circular motion. Therefore, we calculated the standard deviation of the centripetal acceleration in the three acceleration conditions (low, middle, high).

## 5. Results and Discussions

### 5.1. Creative Activity Experiment

Table 1 lists the themes chosen arbitrarily by each subject in Step 2 of the experiment procedure. Because we explained to the subjects that they could perform creative dance freely in Step 1, they considered their feelings in the moment, or the imaginings that came to mind, and therefore the themes had a wide range of variety.

**Figure 8:**
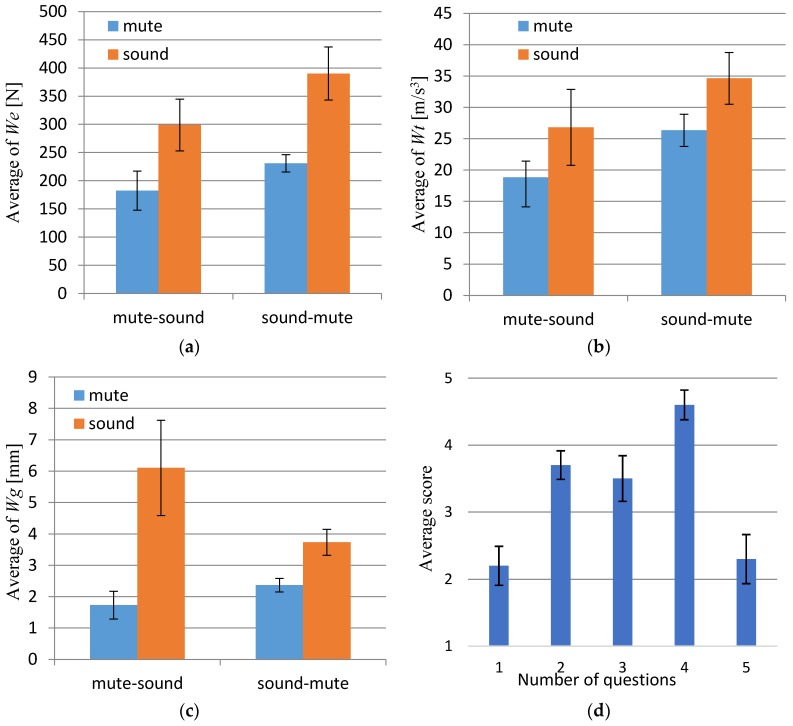
Experimental results. Mute-sound denotes condition “mute” followed by condition “sound”; sound-mute denotes condition “sound” followed by condition “mute”: (**a**) Result of *We*; (**b**) Result of *Wt*; (**c**) Result of Wg; (**d**) Questionnaire results—average score of five (positive) to one (negative).

The experimental results for (a) Weight Effort, *We*, (b) Time Effort, *Wt*, and (c) Grasping evaluation, *Wg* are displayed in Figure 8. The paired *t*-test technique (two-sided) was employed to verify the effectiveness of the proposed interface. It was tested at the 5% significance level. The *t*-test was computed from Figure 8. From the *t*-test, we determined for (a): mute-sound—*We*: t(4) = −3.544, *p* < 0.024 and (a): sound-mute—*We*: t(4) = 4.476, *p* < 0.011; (b): mute-sound—*Wt*: t(4) = −3.254, *p* < 0.031 and (b): sound-mute)—*Wt*: t(4) =3.130, *p* < 0.035; and (c): mute-sound—*Wg*: t(4) = −3.366, *p* < 0.028 and (c): sound-mute—*Wg*: t(4) = 3.462, *p* < 0.026. Therefore, significant differences were observed.

From the results of shown in Figure 8a,b, we can observe that the forces of dance increased and changes in the dance movements became more frequent using TwinkleBall with sound. As an evaluation of the overall body motion, the effectiveness of the proposed interface can be confirmed. Additionally, from the results shown in Figure 8c, both “sound” conditions are higher than “mute” conditions. By using TwinkleBall with sound, it is possible to generate fine grasping motion corresponding to sound change.

Figure 8d is the questionnaire result. Questions No. 1 and 2 provided the performers a subjective evaluation on matching performance to the themes. For Question No. 1, there was a negative evaluation in the case of the “mute” condition. Conversely, for Question No. 2, the score indicates that when the subjects used TwinkleBall with sound, the sound encouraged them to express their imagination through bodily motion. The result of Question 3 shows a positive result with higher rating than the average score of 3. For further improvement of the rating, the delay originating from the interval of communication (35 ms) is a topic of future refinement. Tempo depends on the parameter *v*, as shown in Equation (6). The value of *v* was fixed through these experiments. Since the magnitude of acceleration is affected by the length of the upper arm, tuning of *v* for each individual is considered for future improvement. Question No. 4 scored a positive evaluation. We confirmed that sounds can support the generation of successive motions continuously. Question No. 5 denotes restraint using TwinkleBall. Because it is a negative evaluation, the implication is that the perception of restraint by the interface was not significant.

**Figure 9:**
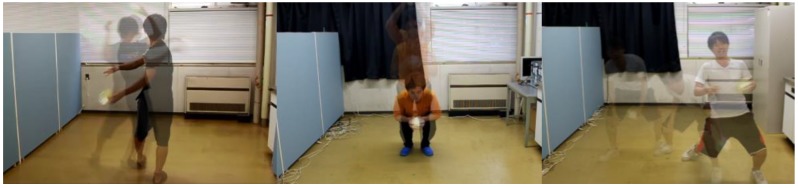
Dance improvisation using TwinkleBall. Subjects provided themes voluntarily: (**left**) “Fun”, (**middle**) “Bamboo shoot” and (**right**) “Gorilla”.

Although the subjects in this experiment chose a variety of themes and the dance motions depended on the theme and personality (Figure 9), we quantitatively observed that the force of dance, variation of dance movements, and grasping movements increased when the dance beginners used TwinkleBall through this experiment. Moreover, we qualitatively confirmed the subjective effectiveness of TwinkleBall to support improvisational creative dance from the results of the questionnaires. Thus, it can be concluded that by using TwinkleBall, it is possible to assist beginner-level dancers perform creative dance.

### 5.2. Movement Accuracy Experiment

**Figure 10:**
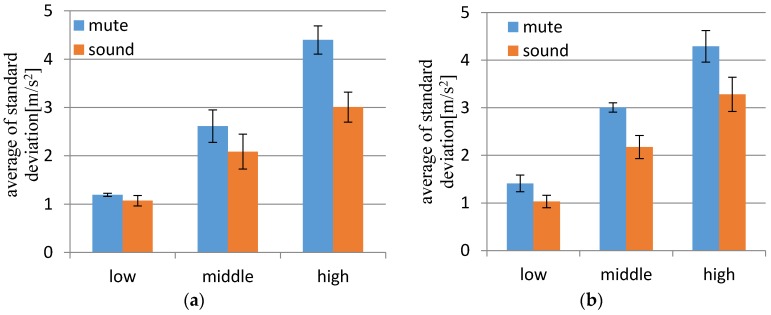
Results for average of standard deviation of detected centripetal acceleration in each target speed: (**a**) condition “mute” followed by condition “sound”; (**b**) condition “sound” followed by condition “mute”.

**Figure 11:**
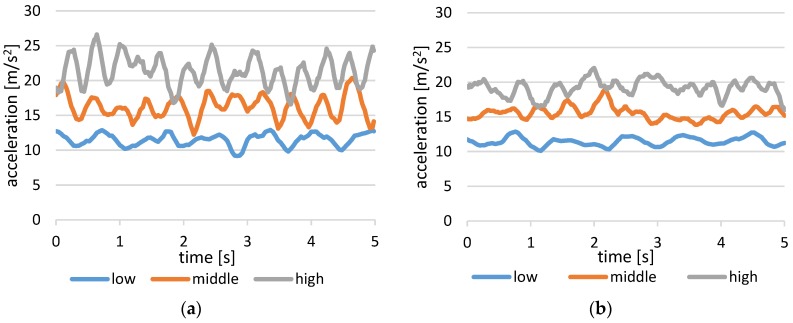
Time sequence results of the magnitude of the accelerations for each target speeds in condition “mute” followed by condition “sound”: (**a**) condition “mute”; (**b**) condition “sound”.

The results of the standard deviation of the centripetal acceleration in each target speed are presented in Figure 10. We compared the values for both the “sound” and “mute” conditions. Additionally, Figure 11 shows an example of the time sequence data of the magnitude of accelerometer data. The deviation of acceleration is larger in “mute” condition than in sound feedback condition, for each of the target speeds.

The paired *t*-test technique (two-sided) was employed for the verification. We tested at the 5% significance level. From the *t*-test in the case of condition “mute” followed by condition “sound” in Figure 10a, we determined low speed: t(4) = 1.201, *p* < 0.296; middle speed: t(4) = 2.785, *p* < 0.049; and high speed: t(4) = 5.587, *p* < 0.005. From the *t*-test in the case of condition “sound” followed by condition “mute” in Figure 10b, we determined low speed: t(4) = −2.130, *p* < 0.100; middle speed: t(4) = −2.835, *p* < 0.047; and high speed: t(4) = −3.272, *p* < 0.031. In the case of both low speeds, no significant difference was observed between the results; however, significant differences can be observed in the cases of middle and high speeds.

In both cases of with and without sound from TwinkleBall, the standard deviation of centripetal acceleration was greater for the greater speed condition. However, the trend of this correlated increase of motion deviation according to target speed was rather mild in the “sound” condition compared to the “mute” condition as indicated by the significant difference in the standard deviation in the middle and high speeds. This could be explained by the different control strategy of human movement. For slower motions, the feedback control is dominant using sensory feedbacks from visual, proprioceptive, and vestibular sensors, continuously correcting the trajectory. Meanwhile, feedforward control is used to generate faster ballistic motions, where the feedback control is overly slow to be fully incorporated. Because the sound generated by the proposed device represented acceleration by rhythmic tempo, it could contribute to stabilizing acceleration in the repeated ballistic feedforward force generation. Thus, TwinkleBall supports the combination of slow and high speed motion and realizes a variety of expression for the dance beginner.

## 6. Conclusions

This paper described the effectiveness of support by the proposed hand-held grasping-type musical interface called TwinkleBall when beginner-level dancers generated physical expression in creative dance. With TwinkleBall, beginners are presented with sounds in real-time that are generated according to their imagined dance performance. Through experiments, we confirmed that these sounds can support successive movement generations continuously during a dance performance. We evaluated in terms of Laban dance movement description, a fine movement expression of grasping motion, and accuracy and repeatability in a task where they produced an imagined circular trajectory by hand. From the results of quantitative measurements and qualitative questionnaires of the creative dance performance, we compared the conditions of TwinkleBall with and without sound. We confirmed that TwinkleBall with sound presentation can increase the force of bodily motion, variation of expression and fine grasping expression, assist creativity, and represent imagined performance accurately for dance beginners.

The device may also be a new tool in brain science research by providing a new task condition that has not been possible without it. Edagawa and Kawasaki [31] measured and analyzed EEG data during rhythmic finger tapping tasks to investigate the brain circuits related to auditory-motor rhythm learning. The new device may for example extend the variety of motion in the researches of this line from simple repetitive finger tasks to complex creative whole body movements.

Future work includes the following perspectives. Firstly, investigation of the after effect in the creative dance task, to evaluate to which extent the enhanced dance performance lasts after detaching or muting the device, can provide practical information for designing a protocol for realistic application of the device to an educational context. In parallel, detailed analysis and evaluation of the enhanced dance performance from a motor control perspective using a 3D motion capture and a physiological measurement might also contribute to this purpose. Though we have focused on beginner level dancers in this study, whole body motion assessment might also contribute to clarification of the level of dancers to which the device is most effective, considering the fact that experienced dancers are better, not only at hand manipulation, but also at whole body coordination during dancing. Secondly, application for music composition using the device as a musical instrument, especially in a co-creative context [32], where multiple persons hold the device in their hands, and dance and generate sounds synchronously according to a theme. Co-creation is effective for live music generation using mobile phone applications [26]. Our device may extend the context of music generation to group dance. The third perspective is rehabilitation for recovery of impaired sensory-motor function after neurological or traumatic damage. Scholz et al. [33] presents possible effectiveness of musical feedback for upper limb rehabilitation after stroke and discusses the advantage of it as inducing motivation of the patients as well as enhancement of sensory-motor learning with the help of the feedback. Playfulness is an important factor of rehabilitation, inducing motivation and active participation of the patients, to which a new technology can contribute [34]. With the enhancement of active motion creativity as well as accuracy, which is shown through this study, our device has possibility of providing a useful tool for rehabilitation.

## Figures and Tables

**Table 1:** Themes in experiments voluntarily chosen by each subject.

Themes				
My neighbor Totoro	Bamboo shoot	Sleepy, but I cannot sleep	Storm	Feeling when it rains
Fun	I want relaxation	Gorilla	Deadline	Running women

## Supplementary Materials

The following are available online at http://www.mdpi.com/1424-8220/17/5/1171/s1, Video S1: grasping; Video S2: Raising arm; Video S3: unconstrained circular hand motion.

## References

[1] Yonezawa M. (2012). The Investigations on the Teachers’ Attitudes to Dance in the Face of Scholastic Requirement of Dance in Middle Schools in Heisei 24 (2012) Academic Year.

[2] Hosotani Y., Tamura N. (2012). Basic Social Skills promoted by dance class at Shikoku University: Focusing on the subjects of a study involving problem-solving. Bull. Shikoku Univ..

[3] National Institute for Educational Policy Research (2011). Creation of Evaluation Criteria, Reference Materials Middle School Health and Physical Education for the Improvement of Device Evaluation Method, etc.

[4] Terayama Y., Hosokawa E. (2011). Teaching and Viewpoints on “Improvised Expression” in the Study of Bodily Expression and Creative Dance. Res. J. JAPEW.

[5] Miyamoto O. (2005). Evaluation of dance performance by students in a creative class. Bull. Middle Sch. Attach. Ochanomizu Univ..

[6] Naugle K.M., Coombes S.A., Cauraugh J.H., Janelle C.M. (2012). Influence of Emotion on the Control of Low-Level Force Production. Res. Q. Exerc. Sport.

[7] Wallbott H.G. (1998). Bodily expression of emotion. Eur. J. Soc. Psychol..

[8] Mendes R.M., Barbosa R.I., Salmon C.E.G., Rondinoni C., Escorsi-Rosset S., Delsim J.C., Barbieri C.H., Mazzer N. (2013). Auditory stimuli from a sensor glove model modulate cortical audiotactile integration. Neurosci. Lett..

[9] Camponogara I., Turchet L., Carner M., Marchioni D., Cesari P. (2016). To hear or not to hear: Sound availability modulates sensory-motor integration. Front. Neurosci..

[10] Laban R., Lawrence F.C. (1947). Effort.

[11] Bartenieff I. (1980). Body Movement: Coping with the Environment.

[12] Yamaguchi T., Kadone H. Supporting creative dance performance by grasping-type musical interface. Proceedings of the 2014 IEEE International Conference on Robotics and Biomimetics (ROBIO).

[13] Sato N., Imura S., Nunome H., Ikegami Y. (2011). The motion characteristic of expert street dancers during performance. Nagoya J. Health, Phys. Fit. Sports.

[14] Sato N., Nunome H., Ikegami Y. Motion characteristics in hip hop dance underlying subjective evaluation of the performance. Proceedings of the 30th Annual Conference of Biomechanics in Sports.

[15] Nakamura A., Tabata S., Ueda T., Kiyofuji S., Kuno Y. Dance training system with active vibro-devices and a mobile image display. Proceedings of the IEEE/RSJ International Conference on Intelligent Robots and Systems.

[16] Yang U., Kim G.J. (2002). Implementation and evaluation of “Just follow me”: An immersive VR-based motion training system. Presence.

[17] Baek S., Lee S., Kim G.J. (2003). Motion retargeting and evaluation for VR-based training of free motions. Vis. Comput..

[18] Fujimoto M., Terada T., Tsukamoto M. A Dance Training System that Maps Self-Images onto an Instruction Video. Proceedings of the International Conference on Advances in Computer-Human Interactions.

[19] Tedenuma M., Maekawa T., Inoue M., Harada I., Iwadate Y., Shiba M. (2003). Development of an Interactive Dance System Suitable to “Kansei (Emotional Expression)” and Confirmation of the Support Effect for Image Transmission. Trans. Virtual Real. Soc. Jpn..

[20] Fujimoto M., Fujita N., Terada T., Tsukamoto M. (2011). Lighting Choreographer: Design and Implementation of a Wearable LED Performance System. Trans. Virtual Real. Soc. Jpn..

[21] Medeiros C.B., Wanderley M.M. (2014). A Comprehensive Review of Sensors and Instrumentation Methods in Devices for Musical Expression. Sensors.

[22] Paradiso J., Hsiao K.Y., Hu E. Interactive music for instrumented dancing shoes. Proceedings of the International Computer Music Conference (ICMC).

[23] Tanaka A. Musical technical issue in using interactive instrument technology with application to the BioMuse. Proceedings of the International Computer Music Conference (ICMC).

[24] Françoise J., Alaoui S.F., Schiphorst T., Bevilacqua T. Vocalizing Dance Movement for Interactive Sonification of Laban Effort Factors. Proceedings of the Designing Interactive Systems.

[25] Landry S., Ryan J., Jeon M. Design issues and consideration for dance-based sonification. Proceedings of the International Conference on Auditory Display.

[26] Oh J., Herrera J., Bryan N.J., Dahl L., Wang G. Evolving the mobile phone orchestra. Proceedings of the New Interfaces for Musical Expression.

[27] Essl G., Müller A. Designing mobile musical instruments and environments with urMus. Proceedings of the New Interfaces for Musical Expression.

[28] Yamaguchi T., Hashimoto S. Grasping interface with photo sensor for a musical instrument. Proceedings of the HCI International.

[29] Yamaguchi T., Kobayashi T., Ariga A., Hashimoto S. TwinkleBall: A wireless musical interface for embodied sound media. Proceedings of the New Interfaces for Musical Expression.

[30] Nakata T., Mori T., Sato T. (2001). Quantitative Analysis of Impression of Robot Bodily Expression based on Laban Movement Theory. J. Robot. Soc. Jpn..

[31] Edagawa K., Kawasaki M. (2017). Beta phase synchronization in the frontal-temporal-cerebellar network during auditory-to-motor rhythm learning. Sci. Rep..

[32] Sawyer R.K. (2006). Education for innovation. Think. Skills Creativity.

[33] Scholz D.S., Rhode S., Großbach M., Rollnik J., Altenmuller E. (2015). Moving with music for stroke rehabilitation: A sonification feasibility study. Ann. N. Y. Acad. Sci..

[34] Shimokakimoto T., Miura A., Suzuki K. (2014). bioToys: Biofeedback toys for playful and self-determined physiotherapeutic activities. Artif. Life Robot..

